# Tenacity of Life in the Infant

**Published:** 1854-06

**Authors:** 


### TENACITY OF LIFE IN THE INFANT.

THE following case illustrative of the remarkable tenacity of life in a human infant, is communicated to us by Dr. JOHN INABNIT, of Oak Grove, Mississippi:—

"A negro girl, about sixteen years of age, near this place, was delivered of a child on the night of the 12th of May; the child was born in the woods, and the mother being alone threw it away. It remained in this situation until the morning of the 14th, perfectly naked, exposed to the sun during the day and to a flood of rain at night. When found it was as cold as a fish, and so remained for several hours. The time was not less than thirty-six hours during which it lay naked, without food in the open air, and yet it is still alive at the end of a fortnight, and I believe, doing well."

